# Polarization-independent surface nanostructuring by femtosecond laser irradiation via microsphere in far field and ambient air

**DOI:** 10.1038/s41377-025-02091-7

**Published:** 2026-02-11

**Authors:** Jingbo Yin, Hao Luo, Tun Cao, Minghui Hong

**Affiliations:** 1 Pen-Tung Sah Institute of Micro-Nano Science and Technology, Xiamen University, Xiamen, 361005 China; 2 Discipline of Intelligent Instrument and Equipment, Xiamen University, Xiamen, 361005 China; 3 School of Optoelectronic Engineering and Instrumentation Science, Dalian University of Technology, Dalian, 116024 China; 4 Innovation Laboratory for Sciences and Technologies of Energy Materials of Fujian Province (IKKEM), Xiamen, 361005 China

**Keywords:** Laser material processing, Lithography

## Abstract

Ultrafast lasers have garnered significant interest in the realm of surface nanofabrication. However, their dynamic electric field distribution is influenced by the polarization direction when pursuing high machining precision, which leads to high polarization dependence of laser nanostructuring. Here, polarization-independent surface nanostructuring is realized on Sb_2_S_3_ thin films by femtosecond laser irradiation via a microsphere in the far field and ambient air. The formation of nanogrooves is ascribed to surface thermal stress during melting, re-solidification, and super-cooling under high-repetition-rate femtosecond laser irradiation. The influence of materials melting and ablation on the electric field distribution during the laser processing is analyzed. In the molten state, the distribution of the electric field remains unaffected by polarization, enabling the realization of polarization-independent nanoprocessing based on the thermal stress induced by a temperature gradient. The feature sizes of surface nanostructures can be precisely adjusted by varying laser fluence, and the minimum size down to approximately 38 nm (λ/27) is achieved. This innovative laser nanostructuring technique, operating in the far field and ambient air, holds considerable promise for advancing next-generation nanofabrication.

## Abstract (web summary)

Polarization-independent Surface Nanostructuring

## Graphical abstract

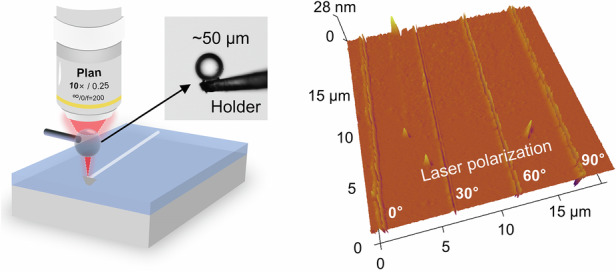

## Introduction

Nanofabrication provides a distinctive platform to explore new applications in the fields of materials science^1^, nanophotonics^2^, and nanobiotechnology^3^. Over the past few decades, processing resolution has been a key driving force for the next generation nanofabrication to create smaller and even smaller features. Electron beam lithography and focused ion beam etching are well known for their ultra-high resolutions^4^. However, as the demand for large-area nanostructures with high resolution continues to rise, achieving this objective using these technologies has become increasingly challenging^5^.

As a non-contact processing technology in ambient air, ultrafast lasers have been widely used in various micro/nano-precision engineering applications because of their broad materials compatibility^6,7^. The focused laser spot diameter, however, is constrained by the optical diffraction limit^8^, which is influenced by both the laser wavelength and the numerical aperture of the objective lens. To overcome this limitation and enhance processing resolution, laser-based sub-diffraction technologies have been developed, broadly categorized into near-field and far-field processing techniques^9^. Among them, the near-field technology relies on the manipulation and control of attenuated evanescent waves, with its propagation range significantly less than the laser wavelength^10^. Limited by extremely short working distance and low throughput, the large-area nanofabrication faces big technical challenges by the near-field processing^11^. Laser irradiation near the ablation threshold of materials can create large-area laser-induced periodic surface structures in the far field^12^, but the controllability of the structure is limited. Laser far-field oxidation combined with ion etching can obtain large-area nanostructures on the silicon surface^13^, but it is difficult to achieve the desired nanostructures. Dielectric microspheres, which can generate sub-wavelength photonic nanojets (PNJ) were used for near-field nano-imaging^14^ and nanofabrication^15^. Recently, there has been increasing interest in using microspheres lifted above the sample surface for non-contact nano-imaging^16^. The integration of non-contact microspheres with ultrafast lasers for far-field nanofabrication presents significant research potential in creating large-area nanostructures^17^.

In addition, laser nanofabrication involves the complex dynamics of interaction between the laser and materials^18^. The dynamic changes in the light field during the processing significantly affect the nanofabrication outcomes, which is another challenge of laser nanofabrication, but the related research is not sufficient. Specifically, the electric field distribution of the subsequent laser pulse is inevitably altered by the surface structure modifications induced by the previous pulses^19^. As a result, the light intensity on the sample surface deviates from the original circularly symmetric Gaussian distribution^20,21^. This phenomenon cannot be ignored when striving for high-precision nanoscale machining. The distribution of the electric field is significantly affected by laser polarization, which is enhanced in a certain direction while weakening in another direction^22^. The non-uniform distribution of the electric field adversely impacts the continuity of laser nanofabrication, leading to a prevalent observation in research that the far-field laser nanoprocessing exhibits a strong polarization dependence^23–25^. Laser scanning along the enhancement direction of the electric field can achieve far-field induced near-field breakdown to obtain the sub-50 nm nanostructures^26^. However, scanning perpendicularly to this direction fails to produce continuous nanostructures owing to significantly reduced laser energy being absorbed for the ablation. Due to the high polarization dependence of laser nanofabrication, the real-time control of laser polarization is necessary^27^. Therefore, addressing the non-uniform distribution of laser intensity during the processing and achieving polarization-independent laser nanofabrication is of paramount importance.

In this work, polarization-independent sub-50 nm structuring is achieved on Sb_2_S_3_ thin films by femtosecond laser irradiation in conjunction with a non-contact microsphere in the far field and ambient air. The dynamics of the electric field distribution during the laser processing are thoroughly analyzed. By maintaining Sb_2_S_3_ in the molten state, the weakening of the electric field can be avoided, thereby enabling the polarization-independent nanostructuring. The nanogrooves formation is attributed to the surface thermal stress, which shows high robustness against changes in laser polarization. Their feature size can be accurately tuned by adjusting laser fluence, with a minimum feature size reduced to approximately 38 nm. This novel approach to achieve polarization-independent laser-induced nanostructures in the far field and ambient air holds significant promise.

## Results

**Fig. 1:**
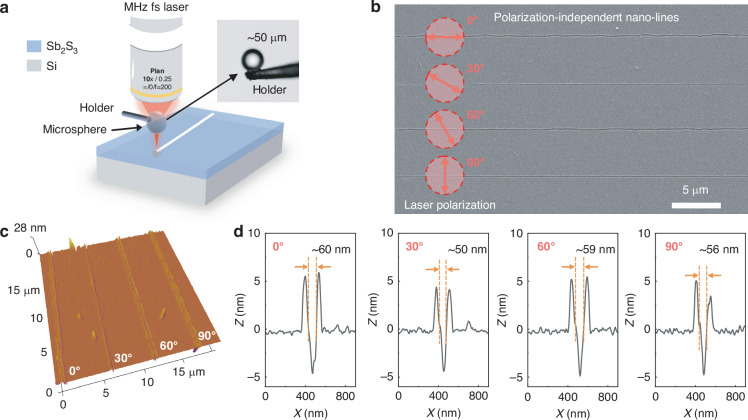
Polarization-independent Surface Nanostructuring. **a** Schematic of polarization-independent nanostructuring in far field via microsphere by MHz femtosecond laser irradiation. **b** SEM and **c** AFM images of polarization-independent nano-lines created at a laser fluence of 0.14 mJ cm⁻² and a scanning speed of 150 μm s⁻¹. **d** Corresponding cross-sectional profiles obtained by AFM measurement

Figure 1a illustrates the polarization-independent surface nanostructuring on Sb_2_S_3_ thin films by femtosecond laser irradiation via a microsphere in far field and ambient air. To attain a more tightly focused laser beam and higher machining accuracy^28^, a femtosecond laser coupled with a microsphere is utilized for the nanoprocessing. Figure S1 investigates the influence of microsphere diameter, microsphere refractive index, and incident light diameter on the PNJ generated by microspheres. A microsphere with a higher refractive index and a smaller radius significantly reduces the full width at half maximum (FWHM) of the PNJ, but at the cost of decreased working distance (WD). To balance the WD and the FWHM of the PNJ, a microsphere at a diameter of 50 μm and a refractive index of 1.5 is employed. As the incident light diameter reduces, the WD increases substantially, whereas the FWHM also decreases markedly. When the incident light diameter falls below 30 μm, the FWHM begins to drop sharply. Thus, the incident light diameter should be ensured to completely cover the upper surface of the microsphere. At this configuration, the microsphere exhibits a focal length of ~31.5 μm, at a corresponding WD of ~6.5 μm and a FWHM of ~920 nm. The focal length is ~30 times the laser wavelength (1030 nm). Thus, the femtosecond laser irradiation via a microsphere works in the optical far field.

Polarization-independent nano-lines are created by high-repetition-rate femtosecond laser irradiation via a microsphere in the far field, as depicted in Fig. 1b. Laser fluence is 0.14 mJ cm⁻², and scanning speed is 150 μm s⁻¹. When the angle between the laser polarization direction and laser scanning direction changes from 0 to 90°, the laser nanoprocessing can always be realized. There is no obvious difference in the obtained nanostructures under different polarization angles, showing polarization-independent surface nanostructuring. In Fig. 1c, the three-dimensional morphology of the nano-line is measured by AFM and the nanostructures show high uniformity. The nanostructuring is conducted under the multi-pulse laser irradiation at a low pulse energy (∼2.8 nJ) and high-repetition-rate (45 MHz). Due to the considerable thermal incubation effect caused by the multiple pulse irradiation, the convex part observed in the AFM image is the molten layer during the laser irradiation. The corresponding cross-sectional profiles of the nano-lines obtained under different polarization angles are measured by AFM, as shown in Fig. 1d. Under different polarization angles, the characteristic size of nanostructures is basically unaffected, and the FWHM of nano-grooves is from 50 to 60 nm. The formation of nano-grooves is interesting. Firstly, its characteristic size is ~1/16 of the focused PNJ (~920 nm), which is due to the nonlinear absorption of the Sb_2_S_3_ film, the top threshold, and the incubation effect of the high-repetition-rate femtosecond laser irradiation. Secondly, the morphology and size of nanostructures are not affected by laser polarization, which is different from the high polarization dependence for most reported laser nanoprocessing strategies. This highly polarization-robust laser nanostructuring is of great significance.

### Mechanisms behind nanostructures formation

**Fig. 2:**
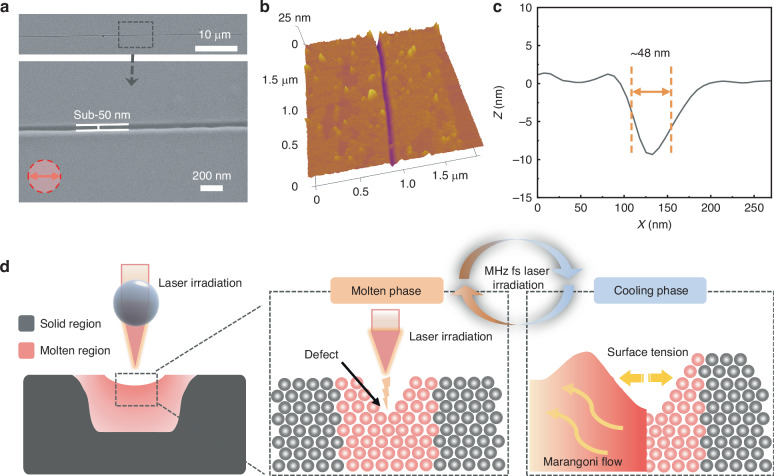
Morphology and Formation Mechanism of Sub-50 nm Nano-line. **a** SEM images of nano-line formed in Sb_2_S_3_ films through microsphere femtosecond irradiation at a laser fluence of 0.12 mJ cm⁻² and a scanning speed of 150 μm s⁻¹. Corresponding **b** three-dimensional morphology and **c** cross-sectional profile of the nano-line obtained by AFM measurement. **d** Schematic of nano-lines formation mechanism in Sb_2_S_3_ films during the molten state under high-repetition-rate femtosecond laser irradiation

A sub-50nm nano-line is observed along the laser scanning path at a laser fluence of 0.12 mJ cm⁻² and a scanning speed of 150 μm s⁻¹, as shown in Fig. 2a. The nano-line demonstrates high structural uniformity across the entire scanning path, exhibiting well-defined boundaries with smooth and continuous edges. The three-dimensional topography and the corresponding cross-sectional images, as measured by AFM, are presented in Fig. 2b, c. By using contact measurement, the depth of the nanogroove is ~10 nm, and the FWHM is ~48 nm. Such high machining accuracy demonstrates a fabrication capability that significantly exceeds the diffraction limit. Under the irradiation at this laser fluence, there is no apparent ablation on the Sb₂S₃ film surface. The characteristic size of the nano-groove is approximately 1/21 of the irradiation laser wavelength (1030 nm), indicating an unusual mechanism behind the nano-grooves formation.

The formation of this nanostructure firstly depends on the focusing of the femtosecond laser going through the microsphere, and the FWHM of PNJ is ~920 nm. Secondly, the band gap of amorphous Sb_2_S_3_ is ~2.05 eV^29^, and the photon energy of the 1030 nm femtosecond laser is ~1.20 eV. The nonlinear effect of two-photon absorption occurs under the femtosecond laser irradiation. As the two-photon absorption is related to the quadratic of laser fluence^30^, the effective intensity distribution is narrowed. Under the action of two-photon absorption, the FWHM of the effective laser focal spot is further reduced to ~680 nm. There is still a gap between the spot size and the achieved size of sub-50nm by the microsphere femtosecond laser irradiation, which indicates that there are other potential mechanisms behind. This whole femtosecond laser processing is carried out under the laser irradiation at a low pulse energy (∼2.4 nJ) and multiple pulses at a high-repetition-rate (45 MHz). This configuration enables the well control of heat accumulation, maintaining it in close proximity to the materials molten threshold, and brings about a top threshold effect and incubation effect^17^. The formation of sub-50 nm structures is primarily attributed to the surface thermal stress during the surface melting, re-solidification, and super-cooling, as well as the incubation effect associated with the high-repetition-rate laser irradiation.

A thermal model is developed to simulate the laser-induced melting, Marangoni effect, and initial stage of nanogroove formation, as shown in Fig. S2 and S3. Based on the simulation and experimental results, the formation mechanism of the nano-groove in the Sb₂S₃ film surface during the molten state under the high-repetition-rate femtosecond laser irradiation is illustrated in Fig. 2d. The energy density of PNJ produced by microspheres is in symmetrical Gaussian distribution, which makes the irradiation center more prone to produce defects^31^. The time interval between the adjacent pulses is ~22 ns. The time from the melting to solidification of Sb₂S₃ is more than 65 ns^32^, so the material remains in the molten state during laser irradiation. As the laser scans, the molten Sb₂S₃ in regions not being irradiated by the laser undergoes cooling and solidification. During the cooling, the surface thermal stress plays a critical role in the formation of nano-grooves. The thermal stress arises from the Marangoni effect, which is caused by the temperature gradient within the laser-induced molten layer^33,34^. In the irradiation of the laser beam with the Gaussian distribution, a temperature gradient is generated from the center to the edge. According to Harkins formula^35^, the surface tension of liquid typically diminishes as temperature rises. The temperature gradient leads to a corresponding gradient of surface tension. Due to this surface tension gradient, Marangoni flow occurs^36^. In Figure S3d, the fluid flows from the area with low surface tension (central high temperature area) to the area with high surface tension (marginal low temperature area)^37^, thus generating a thermal stress. Under the surface thermal stress in the cooling stage, the defects gradually expand. Through multiple pulse irradiation, the materials repeatedly go through melting and cooling processes, and the defects eventually connect along the laser scanning direction to form the nano-grooves.

### Mechanisms behind polarization-independent surface nanostructuring

**Fig. 3:**
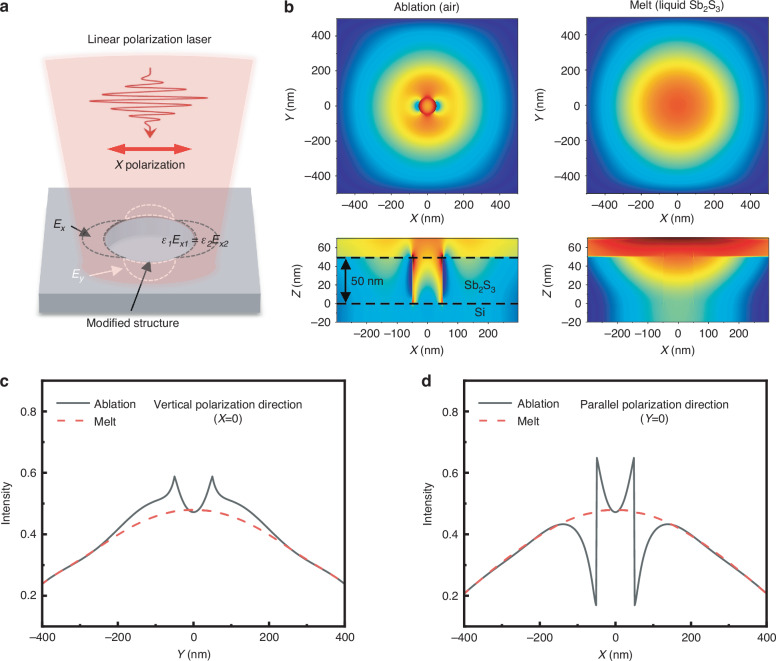
Dynamic Electric Field Distribution in Laser Nanoprocessing. **a** Schematic of laser modified structure influencing the electric field distribution. **b** Simulation of laser electric field distribution in molten and ablation states by FDTD. Specific intensity of electric field distribution in **c** vertical polarization direction (X = 0) and **d** parallel polarization direction (Y = 0) of two states

The potential mechanism affecting the nanoprocessing by laser polarization should be discussed first to avoid polarization influence and create polarization-independent nanostructures. Laser processing is a dynamic process; the electric field distribution of subsequent laser pulses is inevitably modulated by the modified structures caused by the previous pulses^38^. When the subsequent laser pulse is irradiated, the normal component of the electric displacement vector $D$ (parallel to the laser polarization direction ${E}_{P}$) is continuous at the boundary of the modulation structure according to the Gauss Law of electric displacements^20^, as shown in Fig. 3a. Since the electric field intensity and the electric displacement vector satisfy the constitutive relation $D=\varepsilon E$, the electric field near the interface satisfies ${\varepsilon }_{1}{E}_{x1}={\varepsilon }_{2}{E}_{x2}$, where ${E}_{x1}$ and ${E}_{x2}$ respectively represent the electric field (parallel to ${E}_{p}$) near the both sides of the interface, ${\varepsilon }_{1}$ and ${\varepsilon }_{2}$ are dielectric functions on the both sides of the interface. The formula describes the uneven electric field distribution caused by the discontinuous distribution of dielectric function at the edge of the modified structure and leads to polarization-related anisotropy in the laser processing^26,39^. Specifically, under the influence of the modified structure caused by the previous pulses, if the external electric field $({E}_{x2})$ parallel to the polarization direction is enhanced, the external electric field $({E}_{y})$ in the vertical polarization direction is weakened according to the conservation of energy^40^. Therefore, no matter how the dielectric function of the irradiated area changes, there always exists energy weakening in one direction. When pursuing high precision machining, the laser energy is set near the materials threshold^41^. The energy weakening significantly affects processing continuity^42^, rendering it challenging to achieve nanoprocessing in both parallel and vertical polarization orientations simultaneously. To overcome this challenge and realize polarization-independent laser nanofabrication, it is essential to avoid the uneven distribution of the laser field related to polarization during the laser processing.

According to the above analyses, nano-grooves can be formed by the surface thermal stress in the molten state. In addition, there is another case of laser nanoprocessing: by multi-pulse continuous ablation to connect nano-holes and form nano-grooves. When the laser fluence exceeds the ablation threshold, the Sb_2_S_3_ thin film is ablated and removed, so a nano-hole is created in the laser irradiation area during the laser processing. In another case, as the laser fluence is low, Sb_2_S_3_ melts without ablation, creating a nano-molten pool filled with liquefied Sb_2_S_3_. The interval between the adjacent pulses of high-repetition-rate femtosecond laser (~22 ns) is shorter than the thermal relaxation time of Sb_2_S_3_ (~65 ns)^32^, so the Sb_2_S_3_ film is kept in a molten state without solidification during the laser nanoprocessing. As nano-grooves come from the gradual supercooling and reconsolidation of materials from the molten state, the formation of these nano-grooves inevitably lags behind the laser irradiation. In this case, there is only a nano-molten pool in the laser irradiation area during the laser processing.

To further investigate the change of laser field distribution on the Sb_2_S_3_ film surface under two laser nanoprocessing cases, the Finite-Different-Time-Domain (FDTD) simulation is carried out. A Gaussian beam at a wavelength of 1030 nm, a diameter of 900 nm, and an X-polarization direction is simulated as incident light, as shown in Fig. S4. The amorphous Sb_2_S_3_ film thickness is 50 nm while the laser-irradiated nano-hole and nano-molten pool are both 100 nm in diameter and 50 nm in depth. Figure 3b describes the electric field distribution in the two laser nanoprocessing cases. When the Sb_2_S_3_ film is in the ablation state, the subsequent laser is irradiated on the nano-hole. The refractive index of the original amorphous Sb_2_S_3_ film is ~2.8, as illustrated in Fig. S5. Due to the refractive index of air is 1.0, there is a significant dielectric constant discontinuity at the boundary of nano-holes. In the face of subsequent laser pulse irradiation, the energy distribution outside the nano-hole boundary deviates from the original Gaussian distribution. This phenomenon is also supported by experimental observation and theoretical simulation of femtosecond laser irradiation on thin films and transparent solids^22,26^. The electric field distribution in the XY plane reveals that there are two prominent weakened areas located on both edges of the nano-hole, extending along the X-polarization direction. The electric field distribution in the XZ plane demonstrates the significant weakening of the electric field throughout the film area adjacent to both sides of the nano-hole. The laser intensity in the weakened area decreases, thereby having a substantial impact on the continuity of nanoprocessing. When the film is in a molten state, the subsequent laser is irradiated on the nano-molten pool. The liquid refractive index (2.8) is close to the amorphous refractive index^32^, which means that there is no obvious abrupt change in dielectric constant in the laser irradiation area, so the laser energy distribution is basically unaffected by polarization. There is no weakened area around the nano-molten pool, and the electric field distribution retains the original Gaussian symmetrical distribution, thereby ensuring that the entire laser processing is unaffected by the polarization direction. To quantitatively analyze the change of electric field, the specific intensity of the electric field distributions along X (Y = 0) and Y directions (X = 0) are presented in Fig. 3c, d. In the ablation state, there is a significant dielectric constant discontinuity at the boundary of the air-filled nano-hole, so the electric field changes obviously. Due to the electric field redistribution, the normalized intensity outside the nano-hole diminishes from ~0.4 to ~0.1 along X-polarization direction. When the laser intensity approaches the materials ablation threshold, the subsequent laser intensity outside the nano-hole significantly drops below the ablation threshold due to the significant weakening of the electric field. In Fig. S6, with the movement of the Gaussian beam, the weakened area always exists at the hole boundary. This demonstrates that the dielectric constant discontinuity caused by the large refractive index difference leads to the non-uniform distribution of laser intensity, which is inherent and is not affected by the laser irradiation position. This leads to the challenge of creating nanogrooves through continuously ablating nano-holes in the parallel laser polarization direction (X direction). While in the molten state, due to the little change in the refractive index, the dielectric constant at the boundary of the nano-molten pool shows no sudden change, and the electric field strengths in the two directions remain unaffected. Thus, there is no polarization-related anisotropy in the molten state of the laser nanoprocessing.

**Fig. 4:**
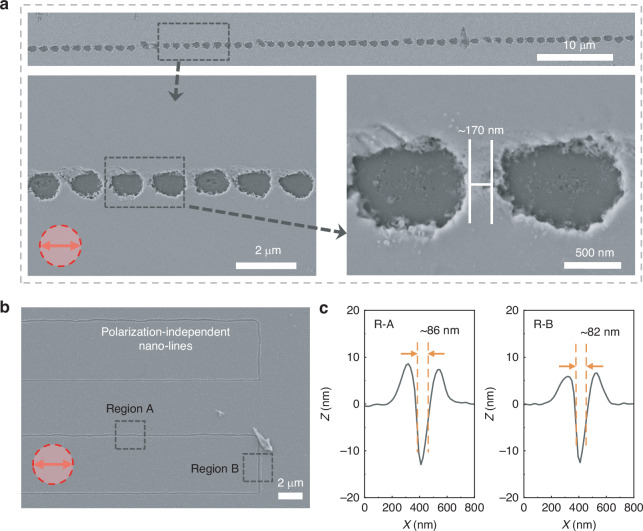
Comparison of Nanostructures Formed in Ablation and Molten States. **a** SEM image of discontinuous ablated nano-holes formed in Sb_2_S_3_ films at a laser fluence of 0.22 mJ cm⁻² and a scanning speed of 150 μm s⁻¹ in the ablation state. **b** SEM image of polarization independent nano-lines created at a laser fluence of 0.18 mJ cm⁻² and a scanning speed of 150 μm s⁻¹ in molten state. **c** Corresponding cross-sectional profiles of regions A and B obtained by AFM measurement

In Fig. 4, the impact of variations in the electric field distribution on the laser nanoprocessing has been demonstrated. At a laser fluence of 0.22 mJ cm⁻² and a scanning speed of 150 μm s⁻¹, the Sb₂S₃ film is ablated. The film enters the ablation stage, wherein rapid materials removal occurs, ultimately leading to the formation of nano-holes on the surface. When the laser scanning direction aligns with the polarization direction, a notable weakened area emerges in the electric field at the nano-hole boundary. The presence of this weakened area disrupts the continuity of processing, ultimately resulting in the formation of discontinuous ablation nano-holes on the film under the laser irradiation, as demonstrated in Fig. 4a. The characteristic size of the nano-holes achieved is approximately 700 nm, at an interval of roughly 170 nm between the adjacent nano-holes. Notably, laser scanning speed is set at 150 µm/s, and laser repetition rate is 45 MHz, implying that within a 170 nm range, there are approximately 5.08 × 10^4^ laser pulses irradiation. Under the irradiation of such a large number of laser pulses, the films are still not continuously ablated to produce a nano-groove, indicating that the non-uniform distribution of laser intensity at the boundaries of ablation nano-holes significantly affects the processing continuity. Hence, the formation of nano-grooves via continuous laser ablation shows strong polarization dependence.

As laser fluence decreases, the Sb₂S₃ film transitions into the molten state. The SEM image confirms the flexible fabrication of the polarization-independent nano-lines by femtosecond laser irradiation at a scanning speed of 150 μm s⁻¹ and a laser fluence of 0.18 mJ cm⁻², as shown in Fig. 4b. There is no obvious ablation on the Sb_2_S_3_ surface, and the formation of nanogroove is attributed to repeated thermal expansion and solidification under the high-repetition-rate laser irradiation. Without changing the laser polarization, nanoprocessing in parallel and vertical polarization directions are both realized. There is no difference in electric fields between parallel and vertical polarizations, so the feature sizes of nano-lines obtained by laser scanning in both directions are basically the same. Meanwhile, even at the scanning corner where the included angle between the laser scanning direction and the polarization direction changes by 90°, the processing remains continuous. The corresponding cross-sectional profiles of the nano-line in regions A and B are measured by AFM, as depicted in Fig. 4c. The FWHM of nanostructures in regions A and B are ~86 and 82 nm, respectively. The feature sizes of nano-lines in the two regions are basically the same, which further proves the high robustness of the processing strategy to the change of polarization direction.

### Laser polarization-independent surface nanostructuring

**Fig. 5:**
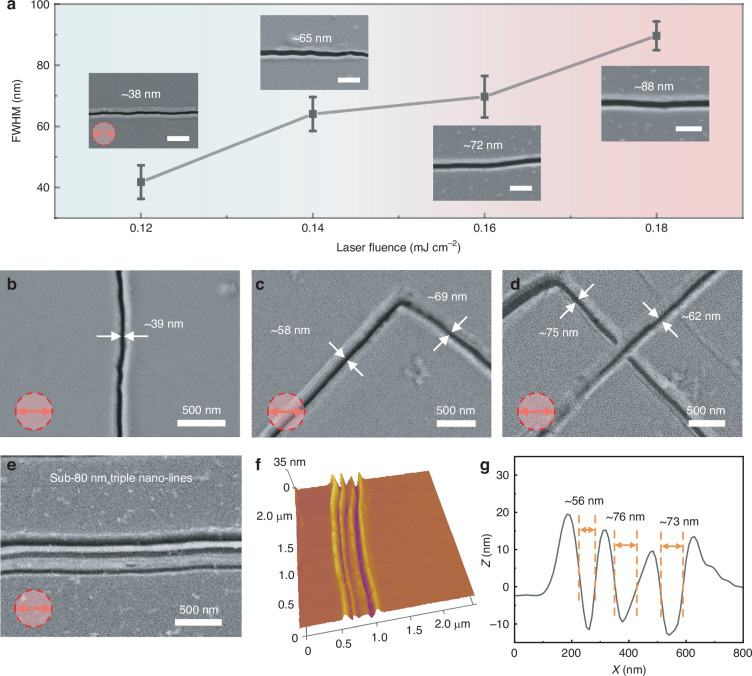
Tunability of Nano-line Feature Sizes and Sub-80 nm Triple Nano-lines prepariton. **a** FWHM of nano-lines made at a scanning speed of 150 μm s⁻¹ and different laser fluences. The scale bar is 500 nm. SEM images of polarization-independent nano-lines made at a scanning speed of 150 μm s⁻¹ and different laser fluences of **b** 0.12, **c** 0.14, and **d** 0.16 mJ cm⁻². **e** SEM and **f** AFM images, as well as **g** corresponding cross-sectional profile of sub-80 nm triple nano-lines created at the laser fluence of 0.16 mJ cm⁻² and scanning speed of 150 μm s⁻¹

The feature size of nano-lines can be turned from sub-100 nm to sub-40 nm by varying laser fluence at a scanning speed of 150 μm s⁻¹, as depicted in Fig. 5a. As laser fluence increases, the FWHM of the nano-lines gradually increases. A higher laser fluence results in a higher surface temperature of the Sb_2_S_3_ films, potentially leading to more molten materials with a higher temperature gradient, which results in enhanced Marangoni flow and higher surface thermal stress, causing the nanogrooves to further grow and subsequently increase the feature size^23^. As the laser fluence is 0.09 mJ cm⁻², the Sb₂S₃ thin film undergoes no melting, only experiencing a phase change from amorphous to crystalline states, as shown in Fig. S7. At the laser fluence of 0.12 mJ cm⁻², the FWHM of the nano-lines is ~38 nm, which is approximately 1/27 of the laser wavelength. As laser fluence increases to 0.18 mJ/cm^2^, the FWHM increases to ~88 nm. The relationship between the FWHM and laser fluence reveals that they are approximately linearly proportional. The feature sizes of the nano-grooves can be precisely adjusted between ~40 and ~100 nm by fine-tuning the laser fluence.

To further validate the polarization independence of this strategy, the influence on nano-lines is investigated when the scanning direction and polarization are oriented at various angles. When the scanning direction is vertical to the polarization, the feature size of the nano-line is ~39 nm at a scanning speed of 150 μm s⁻¹ and a laser fluence of 0.12 mJ cm⁻², as seen in Fig. 5b. When the scanning direction is neither parallel nor vertical to the polarization, the nano-lines obtained at a scanning speed of 150 μm s⁻¹ and varying laser fluences are shown in Fig. 5c, d. Regardless of the angle between laser scanning direction and polarization direction, nanostructures are continuously created. These results further demonstrate that the direction of nanostructures induced by surface thermal stress via microsphere femtosecond laser irradiation is solely dependent on the scanning direction and independent of the laser polarization. The feasibility of creating arbitrary nanostructures by femtosecond laser irradiation via microsphere is also investigated. Nanodots at different sizes are fabricated on Sb_2_S_3_ films by 1.35 × 10^6^ pulse irradiation (exposure time: 30 ms) at different laser fluences, as illustrated in Fig. S8. The feature size of nano-dots is well controlled by adjusting laser fluence. By shortening the exposure time, it is expected to further reduce the feature size. This strategy also enables the free-form writing of nano-patterns with narrow spacing. In Fig. 5e, triple nano-lines with the FWHM of sub-80 nm and period of 150 nm are prepared at the laser fluence of 0.16 mJ cm⁻² and the scanning speed of 150 μm s⁻¹. Nanostructures show high uniformity, and the minimum spacing between nano-lines can be reduced to ~50 nm (λ/21), as shown in Fig. 5f, g.

The fabrication of these nano-lines demonstrates that the microsphere femtosecond laser irradiation can achieve nanostructures with high structural integration. The SEM image of the nanostructures after being stored in ambient air for 80 days is illustrated in Fig. S9. The nanostructures show no obvious morphological change, demonstrating high mechanical and environmental stability. The comparison between this work and established nanostructuring methods in the literature is shown in Table S2, which exhibits the unique characteristics of this strategy. Nanostructures of sub-50 nm feature size, realized through laser direct writing, independent of polarization, have potential applications in the nanofabrication of various optoelectronic devices^43^.

## Discussion

In summary, polarization-independent surface nanostructures are fabricated on Sb_2_S_3_ thin films by femtosecond laser irradiation via a microsphere in the far field and ambient air. By maintaining the material in the molten state, the dynamic weakening of the electric field during the laser processing is avoided, enabling polarization-independent nanostructuring. The formation of nanogrooves is attributed to the surface thermal stress, which results from repeated melting, re-solidification and super-cooling induced by the high-repetition-rate femtosecond laser irradiation. By precisely adjusting laser fluence, the feature sizes of surface nanostructures can be precisely controlled. At a laser fluence of 0.12 mJ cm⁻² and a scanning speed of 150 μm s⁻¹, the minimum size of approximately 38 nm (λ/27) is obtained. This laser nanostructuring strategy, acting in the far field and air ambient environment, brings great promise on the next generation of nanofabrication. Furthermore, the utilization of shorter laser wavelengths, in conjunction with smaller microspheres of a higher refractive index, can lead to a further reduction in the FWHM of PNJ, thereby enhancing machining accuracy potentially down to 10 nm. This strategy also has industrial scalability, and parallel direct writing via microsphere arrays can further improve processing speed and realize large-area nanostructuring. In the future, the potential of this strategy to realize nanostructuring on other materials will also be explored to broaden the work’s applicability.

## Materials and methods

A femtosecond laser (HR-Sci-fs-3 + SHG, HUARAY, China) is applied in our experiment. The laser delivers *t*_*p*_ = 250 fs pulses at *λ* = 1030 nm and a repetition rate of 45 MHz. A microsphere with a diameter of 50 μm (SLGMS, COSPHERIC, America) is secured by a holder and precisely aligned with the laser coaxial microscope system through a three-dimensional translation microstage (with the minimum resolution of 1 μm), as illustrated in Fig. S10. The laser beam is vertically focused onto the microsphere via a 10× objective lens with a numerical aperture (NA) of 0.25 to create photonic nanojets (PNJ). The sample is an amorphous Sb_2_S_3_ film at a thickness of 50 nm, which is deposited on silicon substrate via radio frequency sputtering (RF) at room temperature at a power of 30 W and a chamber base pressure of 5 × 10^-9 ^Torr. The melting point of Sb_2_S_3_ is approximately 800 K. The sample is manipulated using a three-dimensional translation nanostage (NFS100-50PX, OptoSigma, Japan) at the minimum resolution of 10 nm.

The PNJ generated by microspheres and the electric field distribution during the laser processing are simulated using the Lumerical Solutions software, which is based on the Finite-Different Time-Domain (FDTD) method. The surface morphology is characterized by a scanning electron microscope (SEM, SUPRA55 SAPPHIRE, CARL ZEISS, Germany). For three-dimensional morphological characterization and cross-sectional profile analyses, an atomic force microscope (AFM; CYPHER S, ASYLUM RESEARCH, England) is used.

## Supplementary information

Supplementary Information

## Data Availability

The data that support the findings of this study are available from the corresponding author upon reasonable request.
