# Supplementary material for: Polarization-independent surface nanostructuring by femtosecond laser irradiation via microsphere in far field and ambient air
Source: Light Sci Appl. 2026 Feb 11;15:114. doi: 10.1038/s41377-025-02091-7 (PMC12895003; doi:10.1038/s41377-025-02091-7)
Supplement: Supplementary file 1 — Supplementary Information [file 41377_2025_2091_MOESM1_ESM.docx]

**Supplementary Information**

**Polarization-independent Surface Nanostructuring by Femtosecond Laser** **Irradiation via Microsphere in Far Field and Ambient Air**

Jingbo Yin ^a, b^, Hao Luo ^d^, Tun Cao ^d, *^, and Minghui Hong ^a, b, c, *^

^a^ Pen-Tung Sah Institute of Micro-Nano Science and Technology, Xiamen University, Xiamen, 361005, China;

^b^ Discipline of Intelligent Instrument and Equipment, Xiamen University, Xiamen, 361005, China

^c^ Innovation Laboratory for Sciences and Technologies of Energy Materials of Fujian Province (IKKEM), Xiamen 361005, China;

^d^ School of Optoelectronic Engineering and Instrumentation Science, Dalian University of Technology, Dalian 116024, China.

* Correspondence: M. H. Hong, E-mail: elehmh@xmu.edu.cn; T. Cao, E-mail: caotun1806@dlut.edu.cn


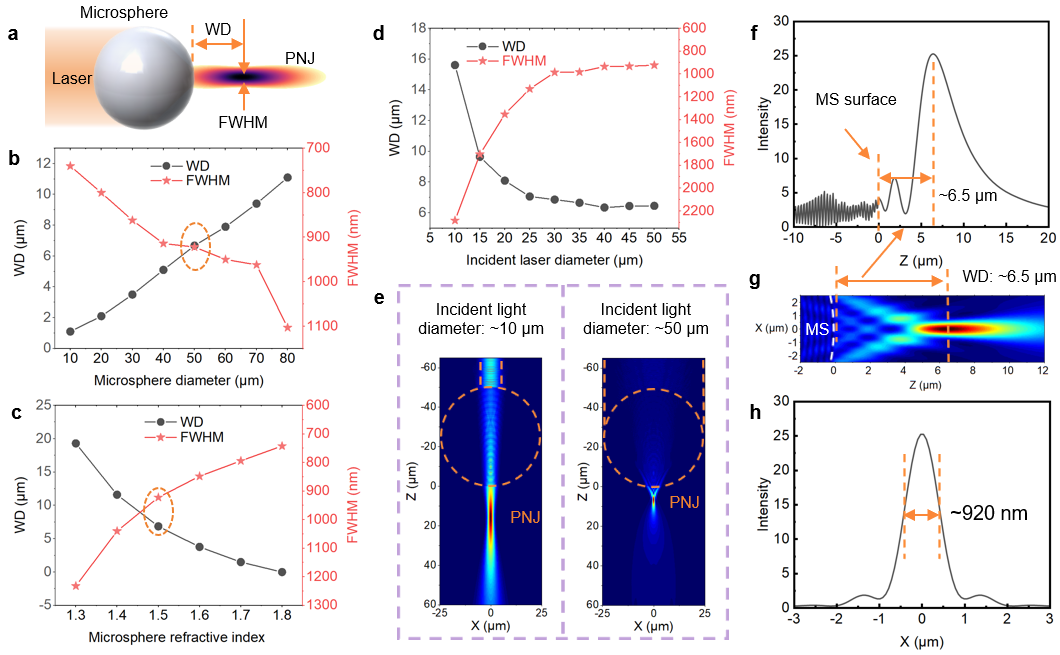


Figure S1 (a) Schematic of PNJ generated by femtosecond laser irradiation through a microsphere. The influence of (b) microsphere diameter, (c) refractive index and (d) incident light diameter on the PNJ generated by microspheres. (e) The electric field distribution of PNJ produced by microspheres at different incident light diameters. The diameter of microsphere is 50 μm and the refractive index is 1.5. (g) The electric field distribution of PNJ with the incident light diameter of 50 μm. The cross-sectional profiles along (f) line X=0, and (h) line Z=6.5 μm (focus position) of (g).


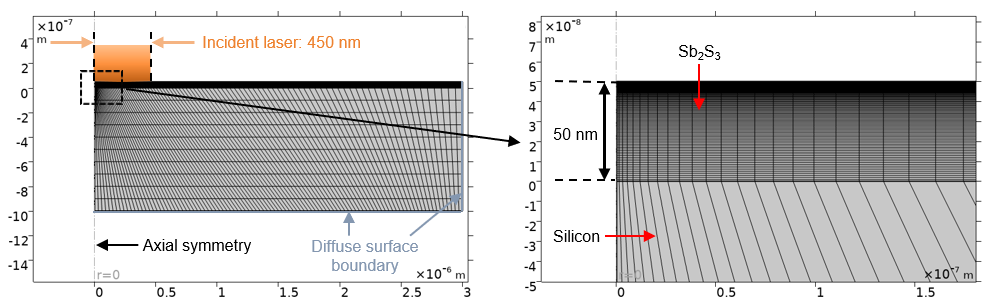
 Figure S2 Mesh and boundary condition of the simulation model.

A thermal model is developed to simulate the laser-induced melting, Marangoni effect and initial stage of nanogroove formation, as shown in Figures S2 and S3. The thermal simulations of the interaction between femtosecond laser and Sb₂S₃ thin films are performed with Heat Transfer in Solid module, Laminar Flow module and 2-dimension Axisymmetric model in COMSOL Multiphysics. The silicon substrate, measuring 3 μm×1 μm, with an overlying Sb₂S₃ thin film of 50 nm×1 μm, is selected for the simulation. The simulation model with detailed labels is shown in Fig. S2.

Under femtosecond laser pulse irradiation, a two-temperature model (TTM) is required to simulate the material heating process. For non-metallic materials, the propagation of femtosecond laser energy occurs in three steps: free carrier excitation described by the photoionization equation, electron absorption of laser energy in the form of photons, and heating of the atomic lattice via electron-phonon interactions. Based on these three processes, the expression for the TTM is given as follows^1^:

$\frac{\partial N_{e}}{\partial t}=\nabla\left(D_{e}\nabla N_{e}\right)+\frac{\alpha I}{h\nu}+\frac{\beta I^{2}}{2hv}-R_{e}$, (S1)

$C_{e}\frac{\partial T_{e}}{\partial t}=\nabla\left(K_{e}\nabla T_{e}\right)-G\left(T_{e}-T_{l}\right)+\frac{\alpha I}{hv}\left(hv-E_{g}\right)+\frac{\beta I^{2}}{hv}\left(2hv-E_{g}\right)+E_{g}R_{g}-3k_{b}T_{e}\frac{\partial N_{e}}{\partial t}$, (S2)

$C_{l}\frac{\partial T_{l}}{\partial t}=\nabla\left(K_{l}\nabla T_{l}\right)-G\left(T_{e}-T_{l}\right)$, (S3)

where$N_{e}$denotes the electron number density,$D_{e}$represents the ambipolar diffusion coefficient, and$R_{e}$stands for the auger recombination electron rate.$\alpha$is the single-photon absorption coefficient, while$\beta$serves as the two-photon absorption coefficient.$I$indicates the input laser intensity,$v$is the laser repetition rate, and$h$is the Planck constant.$k_{b}$is the Boltzmann constant, and$E_{g}$refers to the band gap energy of Sb₂S₃.$C_{l}=\rho C_{\rho}$is the lattice heat capacity,$\rho$is the density of material, $C_{\rho}$is the heat capacity at constant pressure^2^.$K_{l}$is the lattice thermal conductivity.$C_{e}$is the electron heat capacity, which is three orders of magnitude smaller than the lattice heat capacity^2^.$K_{e}=L\sigma T_{e}$is the electron thermal conductivity,$L$is the Lorenz constant,$\sigma$is electrical conductivity^3^.$T_{e}$and$T_{l}$represent the electron temperature and lattice temperature, respectively, and$G$is the electron-lattice coupling coefficient. The supplementary material physical properties beyond the built-in material library in COMSOL are listed in Table S1.

Assuming that the laser beam follows a Gaussian distribution temporally and spatially, the thermal source term can be simplified to the following expression^4^:

$S\left(t,r,z\right)=0.94J_{0}\frac{1-R\left(r,t\right)}{t_{p}\delta}\exp\left[-\frac{r^{2}}{r_{0}^{2}}-2.77\left(\frac{t}{t_{p}}\right)^{2}-\frac{z}{\delta}\right]$ , (S4)

where$r_{0}$and$t_{p}$are beam spot radius and pulse duration, respectively.$J_{0}$is pulse fluence.$R\left(r,t\right)$and$\delta$are the reflectivity and penetration depth of the sample.

Table S1. Physical properties of Sb₂S₃

| Properties | Sb_2_S_3_ |
| --- | --- |
| Melting point | 800 K |
| Density^2^ | 4.6 g cm⁻³ |
| Constant pressure heat capacity^5^ | 121.98 J mol⁻¹ K⁻¹ |
| Electron-photon coupling strength^2^ | 1017 W m⁻³ K⁻¹ |
| Electrical conductivity^2^ | 3.3×10^-8^ S cm⁻¹ |
| Lorenz constant | 2.44×10⁻⁸ W Ω K⁻² |
| Lattice Thermal conductivity^6^ | 0.21 W m⁻¹ K⁻¹ |
| Band gap energy^7^ | 2.05 eV |
| Dynamic viscosity^8^ | 0.1 Pa·s |
| Planck constant | 6.62607015×10^-34^ J·s |
| Boltzmann constant | 1.38 × 10⁻²³ J K⁻¹ |


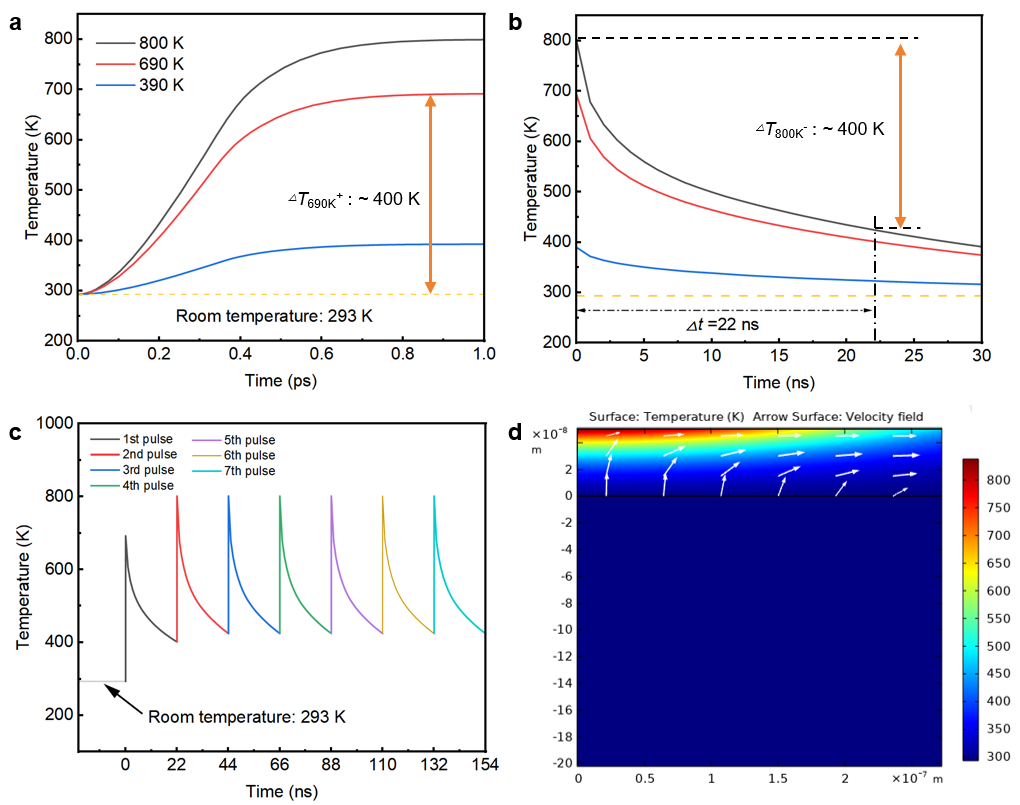


Figure S3 (a) Heating curves of Sb_2_S_3_ for reaching different temperatures. (b) Cooling curves of Sb_2_S_3_ from different temperatures. (c) Temperature variation of Sb_2_S_3_ based on thermal accumulation during high repetition rate femtosecond laser processing. (d) Temperature distribution and melt flow velocity field of Sb_2_S_3_ at 800K.

Figure S3(a) shows the heating time curves of Sb₂S₃ reaching different temperatures under femtosecond laser irradiation. The temperature rising process induced by the previous pulse is completed within 1 ps, and then the material undergoes a cooling process before the next pulse arrives. The laser repetition rate is 45 MHz, and the time interval between adjacent pulses is 22 ns, which is shorter than the thermal relaxation time of the material, as shown in Fig. S3(b). The temperature rise of the material caused by the previous pulse is not completely cooled down before the next pulse arrives. Therefore, there is thermal accumulation between adjacent pulses. Meanwhile, it can be seen that the higher the initial temperature of the cooling process, the faster the cooling rate. The melting point of Sb₂S₃ is ~800 K. When the material is cooled from 800 K, the temperature decreases 400 K in 22 ns. According to the heat accumulation model of high repetition rate femtosecond laser machining proposed in previous literature^9^, the instantaneous temperature rise of the material caused by each pulse is$\Delta T^{+}$, which is proportional to the pulse energy. Then the material cools down$\Delta T^{-}$within 22 ns, and the net temperature rise caused by this pulse is$\Delta T^{+}-\Delta T^{-}$. Due to the thermal accumulation between adjacent pulses, with the increase of the incident pulse, the material temperature continues to rise. While the cooling rate is also proportional to the initial temperature of the cooling process. Therefore, as the material temperature continues to rise, the cooling rate of the material keeps accelerating. When the surface temperature of the material rises to$T_{n}$, the material temperature drop ($\Delta T^{-}$) within 22 ns reaches a balance with the instantaneous material temperature rise ($\Delta T^{+}$) caused by the pulse. At this time, the material temperature does not continue to rise with the increase of the incident pulse, but always cycles between $T_{n}$ and $T_{n}-\Delta T^{-}$.

When the instantaneous temperature rise of the material caused by each pulse is 400 K, the temperature variation of Sb₂S₃ with the number of incident pulses is shown in Fig. S3(c). From the third pulse, the material temperature reaches a dynamic equilibrium. The time from the melting to solidification of Sb₂S₃ is more than 65 ns, so the material does not solidify when it cools down to 400 K. The injection of the next pulse heats the material up to 800 K again, and thus the material can always be maintained in the molten state under high-repetition-rate femtosecond laser irradiation. Due to the non-uniform thermal field of the surface melt, the temperature gradient induces the Marangoni effect. As shown in Fig. S3(d), the surface velocity of the melt has a horizontal component, directed from the high temperature region to the low temperature region. Thus, the Marangoni effect drives the melt to flow from the beam center to the periphery, resulting in a thermal stress directed from the center to the edge. Through multiple pulse irradiations, under the incubation of surface thermal stress, nanogrooves are eventually formed.


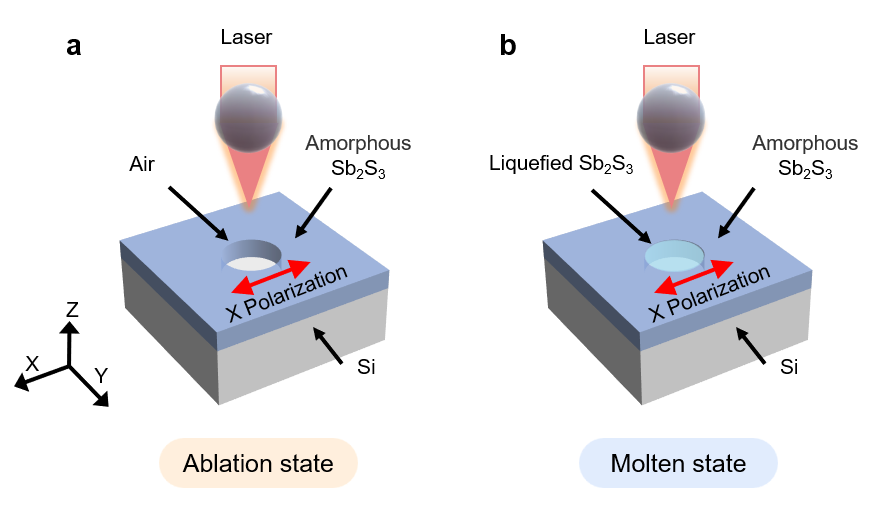


Figure S4 Schematic of simulation model for the change of laser field distribution on the Sb_2_S_3_ film surface under two laser nanoprocessing states: (a) ablation state and (b) molten state.


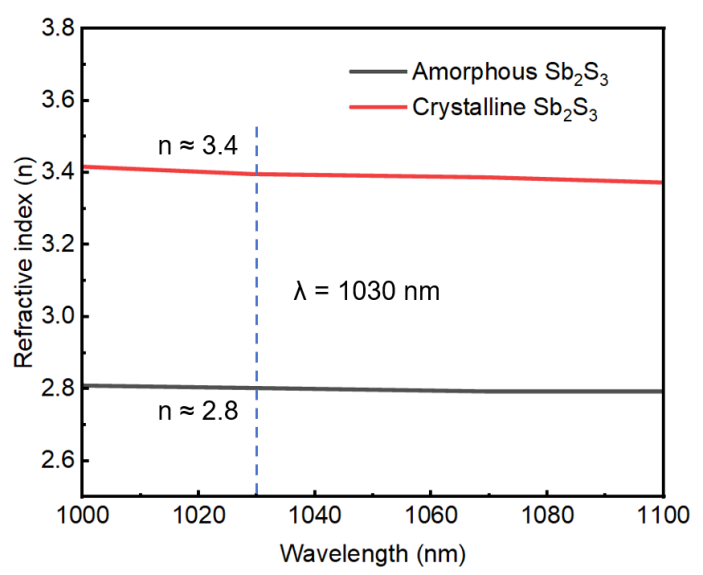


Figure S5 Refractive index of amorphous Sb_2_S_3_ and crystalline Sb_2_S_3_ films measured by an ellipsometer in the spectral range from 1000 to 1100 nm.


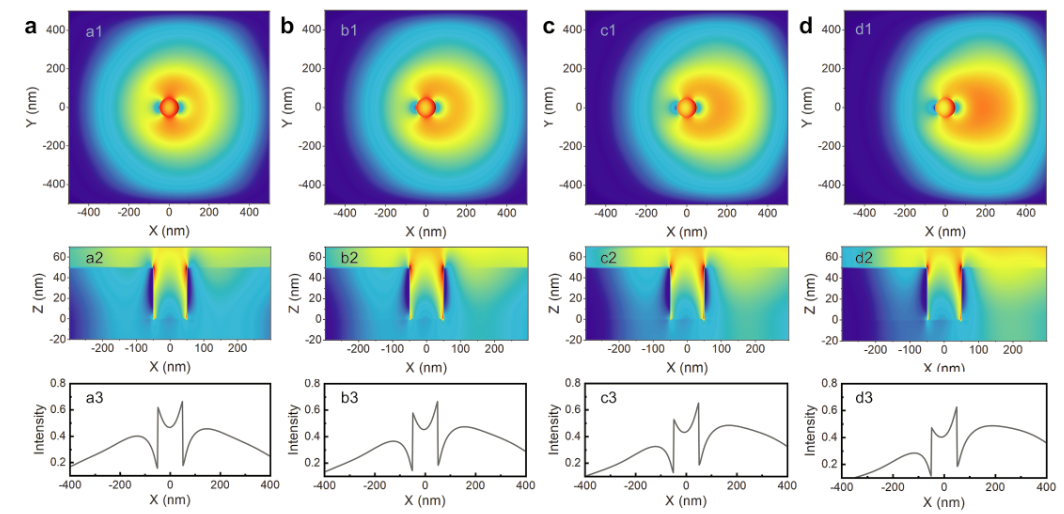


Figure S6 Simulation of laser electric field distribution by FDTD method, when the Gaussian beam deviates from the center of the nano-hole by (a) 50, (b) 100, (c) 150, and (d) 200 nm.


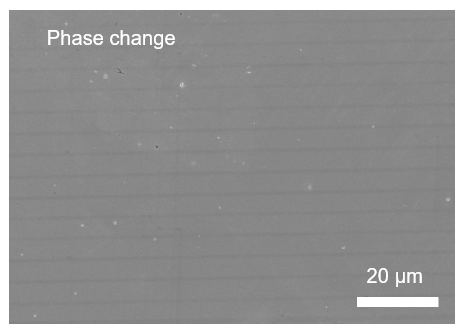


Figure S7 SEM images of nano-phase change region in Sb_2_S_3_ films through microsphere femtosecond irradiation at a scanning speed of 150 μm s⁻¹ and a laser fluences of 0.09 mJ cm⁻².


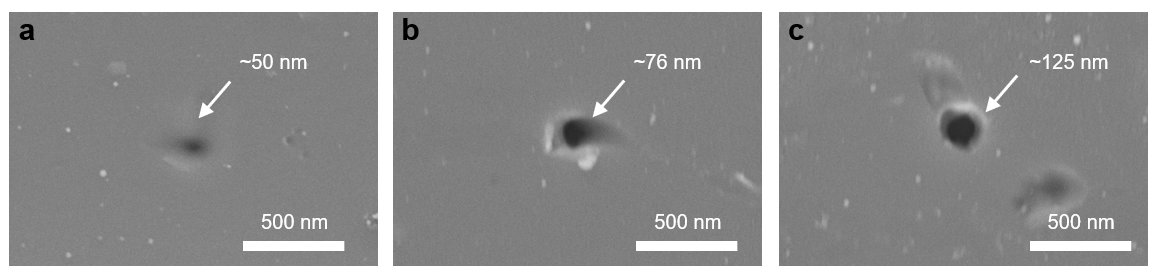


Figure S8 SEM images of the nanodots with different sizes on Sb_2_S_3_ films by 1.35 ×10^6^ pulse number irradiation (exposure time: 30 ms) at different laser fluences of (g) 0.12, (h) 0.14, and (i) 0.18 mJ/cm^2^.


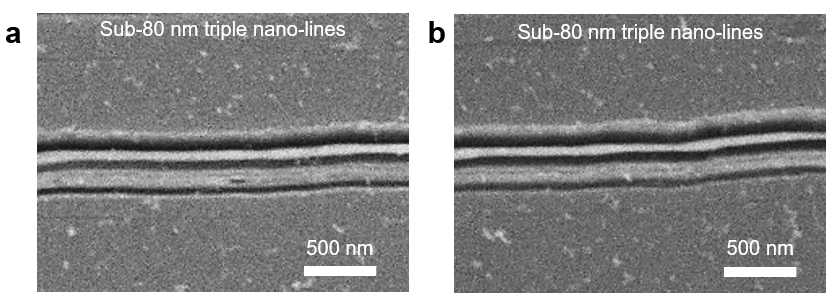


Figure S9 SEM image of the nanostructures (a) before and (b) after stored in ambient air for 80 days.

Fig. S10 shows the schematic of polarization-independent surface nanostructuring on Sb_2_S_3_ thin films by non-contact microsphere femtosecond laser irradiation. The femtosecond laser at a wavelength of 1030 nm and a pulse duration of 250 fs is integrated with a 451 nm light source within the same optical path using two beam splitters (BS)，while the average ratios of reflectance and transmission are approximately 50∶50 at both 1030 and 451 nm. The laser polarization direction is adjusted by a half-wave plate before focusing. The microsphere at a diameter of 50 μm is secured by a holder and precisely aligned with the laser coaxial microscope system through a three-dimensional translation microstage (with the minimum resolution of 1 μm). The sample is manipulated using a three-dimensional translation nanostage (NFS100-50PX, OptoSigma, Japan) at the minimum resolution of 10 nm. The charge-coupled device (CCD) is used in conjunction with the objective lens to observe the laser focus position, ensuring that the laser beam passes through the microsphere accurately.


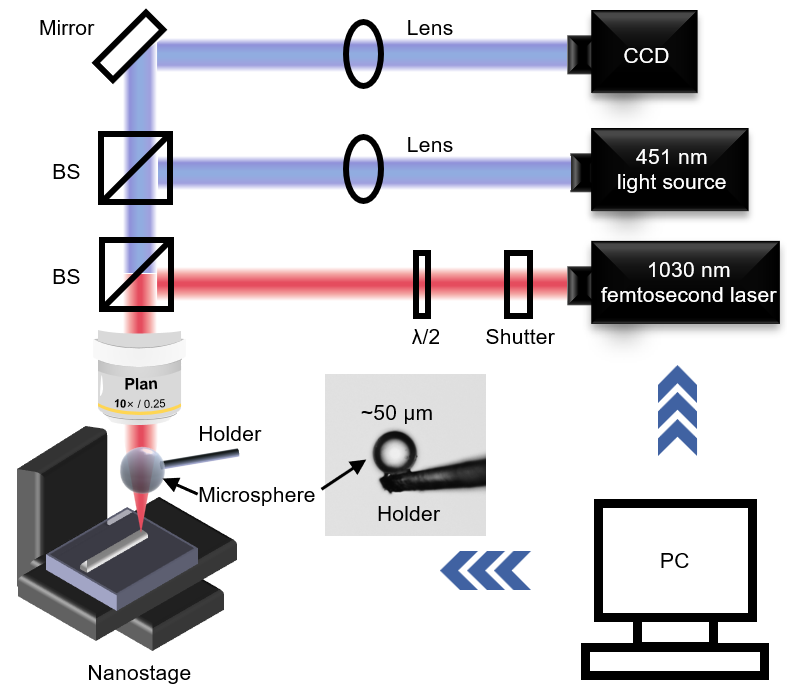


Figure S10 Schematic of polarization-independent nanostructuring via non-contact microsphere femtosecond laser irradiation.

Table S2 Comparison between this work and established nanostructuring methods in the literature.

| Light source | System cost | Working environment | Resolution/wavelength | Processing speed | Polarization effect | Reference |
| --- | --- | --- | --- | --- | --- | --- |
| EUV (13.5 nm) | High | Vacuum | ~5 nm (λ/2.7) | High | \ | 10 |
| EUV (13.5 nm) | High | Vacuum | ~7 nm (λ/1.9) | High | \ | 11 |
| Fs laser (800 nm) | Medium | Near Field and Ambient air | ~50 nm (λ/26) | Low | Dependent | 12 |
| Ns laser (532 nm) | Medium | Near Field and Ambient air | ~151 nm (λ/3.5) | Low | Dependent | 13 |
| Ns laser (1064 nm) | Low | Far Field and Ambient air | ~30 nm (λ/26) | High | Dependent | 14 |
| Fs laser (800 nm) | Low | Far Field and Ambient air | ~20 nm (λ/40) | High | Dependent | 15 |
| Fs laser (1030 nm) | Low | Far Field and Ambient air | ~38 nm (λ/27) | High | Independent | This work |

# References

1 Liu J, Wu M, Sun Z, Zhang Q, Zhu Y, Fu Y. The picosecond laser ablation mechanism of monocrystalline silicon by coupling two-temperature model (TTM)-Molecular dynamic (MD). *Appl Surf Sci* 2024; **661**: 160022.

2 Liu H, Dong W, Wang H, Lu L, Ruan Q, Tan YS *et al.* Rewritable color nanoprints in antimony trisulfide films. *Sci Adv* 2020; **6**: eabb7171.

3 Kivinen P, Savin A, Zgirski M, Törmä P, Pekola J, Prunnila M *et al.* Electron–phonon heat transport and electronic thermal conductivity in heavily doped silicon-on-insulator film. *J Appl Phys* 2003; **94**: 3201–3205.

4 Liu H, Lin W, Lin Z, Ji L, Hong M. Self-Organized Periodic Microholes Array Formation on Aluminum Surface via Femtosecond Laser Ablation Induced Incubation Effect. *Adv Funct Mater* 2019; **29**: 1903576.

5 Ben Nasr T, Maghraoui-Meherzi H, Kamoun-Turki N. First-principles study of electronic, thermoelectric and thermal properties of Sb_2_S_3_. *J Alloy Compd* 2016; **663**: 123–127.

6 Aryana K, Kim HJ, Islam MR, Hong N, Popescu C-C, Makarem S *et al.* Optical and thermal properties of Ge_2_Sb_2_Te_5_, Sb_2_Se_3_, and Sb_2_S_3_ for reconfigurable photonic devices. *Opt Mater Express* 2023; **13**: 3277–3286.

7 Dong W, Liu H, Behera JK, Lu L, Ng RJH, Sreekanth KV *et al.* Wide Bandgap Phase Change Material Tuned Visible Photonics. *Adv Funct Mater* 2019; **29**: 1806181.

8 Kassem M, J. Benmore C, Tverjanovich A, Usuki T, Khomenko M, Fontanari D *et al.* Glassy and liquid Sb_2_S_3_: insight into the structure and dynamics of a promising functional material. *J Mater Chem C* 2023; **11**: 4654–4673.

9 Kerse C, Kalaycıoğlu H, Elahi P, Çetin B, Kesim DK, Akçaalan Ö *et al.* Ablation-cooled material removal with ultrafast bursts of pulses. *Nature* 2016; **537**: 84–88.

10 Giannopoulos I, Mochi I, Vockenhuber M, Ekinci Y, Kazazis D. Extreme ultraviolet lithography reaches 5 nm resolution. *Nanoscale* 2024; **16**: 15533–15543.

11 Mojarad N, Hojeij M, Wang L, Gobrecht J, Ekinci Y. Single-digit-resolution nanopatterning with extreme ultraviolet light for the 2.5 nm technology node and beyond. *Nanoscale* 2015; **7**: 4031–4037.

12 Ueno K, Takabatake S, Nishijima Y, Mizeikis V, Yokota Y, Misawa H. Nanogap-Assisted Surface Plasmon Nanolithography. *J Phys Chem Lett* 2010; **1**: 657–662.

13 Wang X, Mei X, Yin H, Wang Z, He X, Cui J. Locally excited surface plasmon polaritons (SPPs) induced scanning near-field optical lithography on Ag nano-film. *Opt Laser Technol* 2025; **189**: 113122.

14 Lin Z, Ji L, Hong M. Approximately 30 nm Nanogroove Formation on Single Crystalline Silicon Surface under Pulsed Nanosecond Laser Irradiation. *Nano Lett* 2022; **22**: 7005–7010.

15 Li Z-Z, Wang L, Fan H, Yu Y-H, Chen Q-D, Juodkazis S *et al.* O-FIB: far-field-induced near-field breakdown for direct nanowriting in an atmospheric environment. *Light Sci Appl* 2020; **9**: 41.
